# Improved *n*-butanol production by a non-acetone producing *Clostridium pasteurianum* DSMZ 525 in mixed substrate fermentation

**DOI:** 10.1007/s00253-014-5588-8

**Published:** 2014-03-02

**Authors:** Wael Sabra, C. Groeger, P. N. Sharma, An-Ping Zeng

**Affiliations:** 1 Institute of Bioprocess and Biosystems Engineering, Hamburg University of Technology, Denickestr.15, 21073 Hamburg, Germany; 2 Microbiology Department, Faculty of Science, Alexandria University, Alexandria, Egypt

**Keywords:** Butanol production, *Clostridium pasteurianum*, ABE fermentation, Mixed substrate fermentation

## Abstract

The kinetics of growth, acid and solvent production in batch culture of *Clostridium pasteurianum* DSMZ 525 were examined in mixed or mono-substrate fermentations. In pH-uncontrolled batch cultures, the addition of butyric acid or glucose significantly enhanced *n*-butanol production and the ratio of butanol/1,3-propanediol. In pH-controlled batch culture at pH = 6, butyric acid addition had a negative effect on growth and did not lead to a higher *n*-butanol productivity. On the other hand, mixed substrate fermentation using glucose and glycerol enhanced the growth and acid production significantly. Glucose limitation in the mixed substrate fermentation led to the reduction or inhibition of the glycerol consumption by the growing bacteria. Therefore, for the optimal growth and *n*-butanol production by *C. pasteurianum*, a limitation of either substrate should be avoided. Under optimized batch conditions, *n*-butanol concentration and maximum productivity achieved were 21 g/L, and 0.96 g/L × h, respectively. In comparison, mixed substrate fermentation using biomass hydrolysate and glycerol gave a *n*-butanol concentration of 17 g/L with a maximum productivity of 1.1 g/L × h. In terms of productivity and final *n*-butanol concentration, the results demonstrated that *C. pasteurianum* DSMZ 525 is well suitable for *n*-butanol production from mixed substrates of biomass hydrolysate and glycerol and represents an alternative promising production strain.

## Introduction

Before the 1950s, acetone-butanol-ethanol (ABE) fermentations using Clostridia ranked second to ethanol in importance and scale of bioproduction processes. The ABE fermentation declined or was even shut down because of increased substrate costs and the availability of much cheaper, petrochemically derived butanol (Zverlov et al. 2006). However, in recent years, there has been a renewed interest in this fermentation, which has led to a large number of studies on the metabolism and genetics of solventogenic Clostridia and on the improvement of fermentation and product recovery technologies (Garcia et al. 2011). Currently, there are still three major hurdles for fermentative *n*-butanol to compete with the petroleum-based one (Biebl 2001; Jang et al. 2012; Lee et al. 2012; Tracy 2012). These include (1) high cost of substrates: molasses, as typically used as a substrate in the past, may account for about 50 % of the final cost of the product (Vandecasteele and Marchal 1993); (2) low final product concentrations due to limited bacterial tolerance and (3) high product recovery costs due to the low concentration of *n*-butanol produced. Recent interest in the production of biobutanol from biomass has led to the reexamination of the *n*-butanol fermentation. Advances in strain development, integrated fermentation and in situ product removal processes have resulted in a dramatic reduction of process streams, reduced *n*-butanol toxicity to the fermenting microorganisms, improved substrate utilization and overall bioreactor performance. Therefore, a possible reintroduction of large-scale ABE fermentation appears increasingly feasible (Jesse et al. 2002; Qureshi et al. 2006, 2007, 2008; Lee et al. 2012; Liu et al. 2013). Nevertheless, the cost of *n*-butanol recovery still remains high. Significant energy savings can be achieved if the concentration of *n*-butanol in the fermentation broth is increased.

In order to realize industrial-scale *n*-butanol fermentation, an efficient process based on a high-performance *n*-butanol producer utilizing inexpensive carbon substrate such as biomass hydrolysate (BH) from cellulosic materials is desired. Beside, glycerol as a byproduct from biodiesel production has recently also attracted much attention as a potential substrate for bio-based production of chemicals and fuels. Their abundance and cost competitiveness make both substrates excellent candidates for *n*-butanol production. The mostly studied microorganism for biological production of *n*-butanol is *Clostridium acetobutylicum*, and ABE fermentation by using this microorganism was the basis for *n*-butanol production on an industrial scale for a long period of time. However, *C. acetobutylicum* is unable to grow on glycerol as a sole carbon source, since it cannot efficiently reoxidize the excess NADH generated in glycerol catabolism (Patakova et al. 2013). *Clostridium pasteurianum*, on the other hand, is known as a potentially promising producer of *n*-butanol. This microorganism normally does not produce acetone and ethanol, but organic acids such as acetic and butyric acids as major fermentation products from sugars. It can also produce 1,3-propanediol (1,3-PDO) when grown on glycerol (Biebl 2001; Jensen et al. 2012a, b). Biebl (2001) first reported the production of 17 g/L *n*-butanol in batch cultures with *C. pasteurianum* grown in semi-synthetic media containing glycerol as the sole carbon source. However, a great variation in the product formation was observed in batch cultures: i.e. either *n*-butanol or 1,3-PDO could be produced as the major product under equal or slightly different conditions. A weak pathway regulation and the existence of multiplicity have been assumed by Biebl (2001). Recently, Moon et al. (2011) and Khanna et al. (2013a) optimized the medium compositions to favour *n*-butanol or 1,3-PDO production in free and immobilized cultures of *C. pasteurianum*, respectively. Kao et al. (2012) studied the co-fermentation of glucose and glycerol by using the strain *C. pasteurianum* CH4. The *n*-butanol production was increased from 11 g/L in a medium containing sole glycerol to 13.3 g/L in a medium containing both glucose and glycerol. The maximum *n*-butanol productivity obtained under optimal conditions was 0.28 g/L × h in this co-fermentation process. The productivity can be significantly improved by in situ *n*-butanol removal, as demonstrated by Jensen et al. (2012a, b) using gas stripping to remove *n*-butanol in glycerol fermentation with *C. pasteurianum* DSMZ 525. With gas stripping during the fermentation, these authors could achieve *n*-butanol productivity as high as 1.3 g/L × h. Using the same *C. pasteurianum* strain (DSMZ 525) but without the in situ *n*-butanol removal, we report in this work an optimized *n*-butanol production process using mixed substrates. It is shown that glucose as a co-substrate in the glycerol fermentation with *C. pasteurianum* can significantly enhance the cell growth and consequently the *n*-butanol productivity. A maximum productivity of 0.96 g/L × h and a final *n*-butanol concentration of 21.5 g/L were reached. This represents the highest *n*-butanol titer ever reached in a normal suspension culture of Clostridia. The use of BH was also evaluated in the co-substrate bioprocess for *n*-butanol production.

## Materials and methods

### Microorganism and medium

*C. pasteurianum* DSMZ 525 was cultivated anaerobically at 35 °C without shaking. Stock cultures were maintained on Reinforced Clostridial Medium (RCM, Oxoid Deutschland GmbH, Wesel, Germany) and preserved using glycerol 20 % (*v*/*v*) at −80 °C. Liquid cultivation was done in serum bottles containing either the RCM or the production media. Resazurin (7-hydroxy-10-oxidophenoxazin-10-ium-3-one) was added at a concentration of 1 mg/L as a redox indicator for anaerobiosis.

RCM inoculated from a cryoculture was left to grow at 35 °C for 18–20 h and then used as inocula for the production medium. The standard production medium for batch cultures contained the following ingredients in 1 L of distilled water (modified from Biebl 2001): glycerol, varied; glucose, varied; K_2_HPO_4_, 0.5 g; KH_2_PO_4_, 0.5 g, MgSO_4_ · 7H_2_0, 0.2 g; (NH_4_)_2_SO_4_, 5 g; CaCl_2_ 2H_2_O, 0.02 g, FeSO_4_ 7H_2_O, 0.01 g; cysteine HCl, 0.3 g; resarzurin, 0.005 g; 2 mL of trace element solution SL7 and 1 g yeast extract. In anaerobic bottles, the medium pH was adjusted to 7 with 1 N NaOH and CaCO_3_ was added at a concentration of 2 g/L.

Uncontrolled batch experiments were done using 100-mL serum bottles filled with 50 mL medium containing 25 g/L glycerol and the different co-substrates. The pH was adjusted and the medium was boiled and cooled under nitrogen to ensure anaerobic condition and autoclaved. Glucose was autoclaved separately in anaerobic bottles with nitrogen as headspace and added after autoclaving. Experiments were conducted in triplicate, and culture was inoculated with 5 % (*v*/*v*) of RCM growing cultures and incubated for 5 days.

Batch and fed-batch cultivations were carried out in a pH-controlled 2-L stirred tank bioreactor (Bioengineering) with a working volume of 1.5 L. After sterilization, the medium in the bioreactor was flushed with sterile oxygen-free nitrogen gas until room temperature was reached. Glucose was autoclaved separately and was added together with the sterile cystein HCl and FeSO_4_ solution and inoculated immediately. Spruce BH (Borregard, Norway) was also used in co-substrate fermentation with glycerol. The enzymatic hydrolysis of spruce was done without buffer. The hydrolysate was then heated at 80 °C for 15–20 min to inactivate the enzymes. The supernatant was removed and filtered with a centrifuge with filter bag. The sample was then concentrated by vacuum evaporation at 60 °C. The concentrated hydrolysate contained 550 g/L glucose and 35 g/L xylose and was autoclaved separately. Flushing with nitrogen was stopped after inoculation, and the bacteria were grown under their own produced gases. The pH was adjusted to 6 by automatic addition of 5 N KOH.

### Analytical methods

Cell concentration was measured turbidometrically at 600 nm and correlated with cell dry weight determined directly. In the cell-free supernatants, the concentrations of glucose, glycerol, butanol, 1,3-PDO, ethanol, acetic, butyric and lactic acids were determined by HPLC using an Aminex HPX-87H column (300 × 7.8 mm) and detection was assessed by refractive index and ultraviolet detectors. The HPLC operating conditions were as follows: mobile phase, 5 mM H_2_SO_4_; flow rate, 0.6 mL/min and temperature, 60 °C. Acetone was detected with an Agilent headspace gas chromatography (6,890 N) equipped with a Stabiwax DA capillary column (60 m × 320 μm) and a FID detector. Helium was used as a carrier gas (100 kPa). The analysis was run at 50 °C for 1 min and then 15 °C/min till 200 °C for 5 min.

## Results

### pH-uncontrolled cultures of *C. pasteurianum* with different co-substrates

**Fig. 1:**
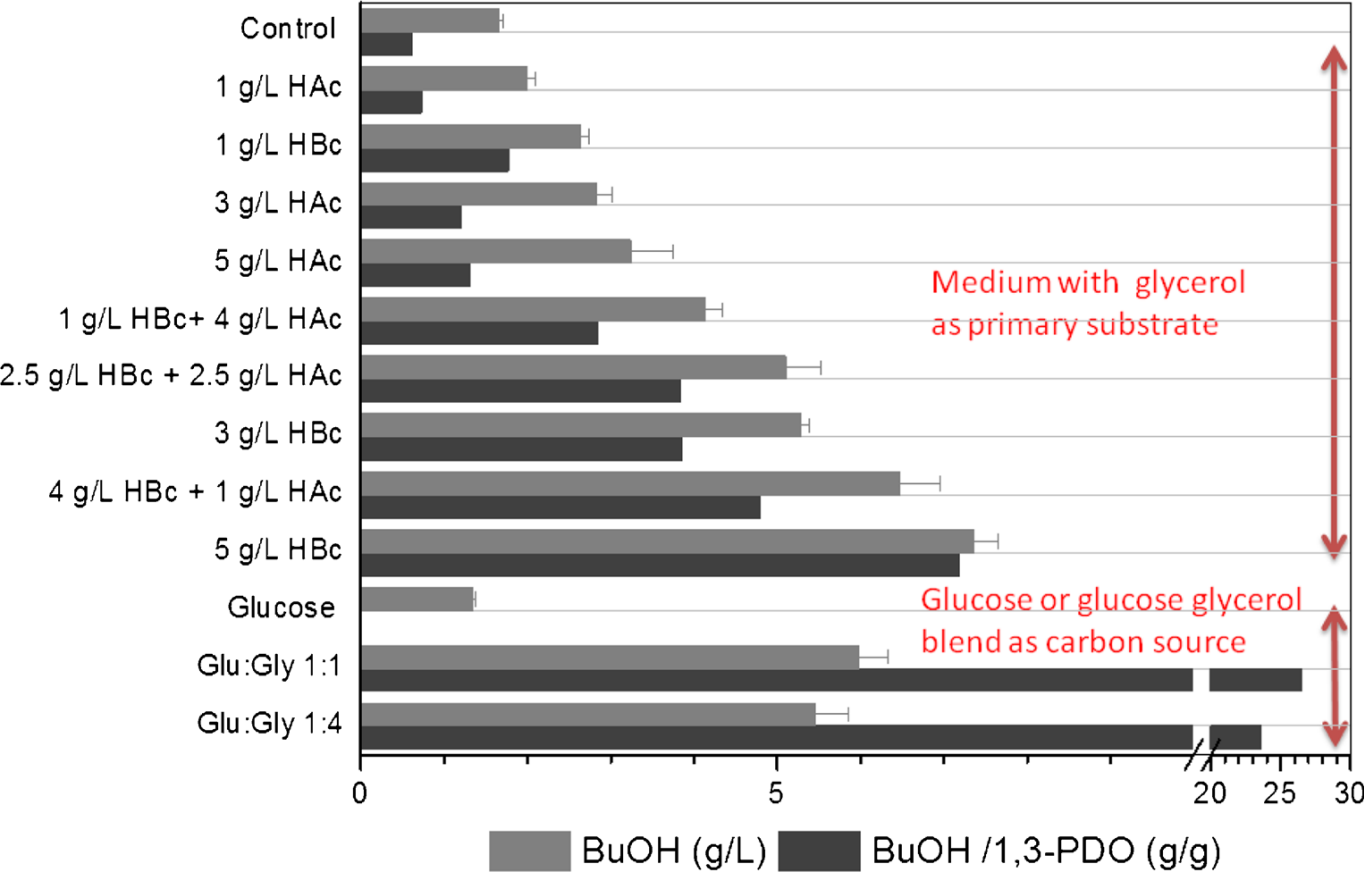
The effects of acetic (*HAc*), butyric acid (*HBc*) and glucose addition on butanol (*BuOH*) formation from glycerol

Results of growth and product formation of *C. pasteurianum* in anaerobic bottles supplemented with various combinations of co-substrates are presented in Fig. 1. The pH of the medium was adjusted to pH 7, and stock solutions of organic acids were neutralized (pH 7), sterile filtered and added to the anaerobic bottles at different concentrations. It was found that the addition of increasing amounts of organic acids, and especially, butyric acid enhanced the production of butanol. A maximum of 7 g/L *n*-butanol was produced by the strain grown on glycerol supplemented with 5 g/L butyric acid, compared to 1.85 g/L *n*-butanol in mono-substrate fermentation. The butanol/1,3-PDO ratio also increased steadily with increasing butyric acid concentration. Butyric acid concentrations above 5 g/L resulted in diminished cell growth. On the other hand, in bottles with glucose or blend of glucose and glycerol, acid production increased significantly and was five to eightfold higher compared to growth on glycerol. Since no natural pathway exists for a direct production of 1,3-PDO from glucose, the butanol/1,3-PDO ratio increased significantly in bottles with co-substrates. The gases produced during bacterial growth resulted in increased pressure in the anaerobic bottles. Under these conditions, the glycerol initially added (25 g/L) was never completely consumed even after 110 h.

### Butyrate addition and *n*-butanol production in pH-controlled bioreactors

The co-substrate fermentation with *C. pasteurianum* was further studied in controlled bioreactor to maximize the *n*-butanol production. In a pH-controlled batch culture with a medium containing glycerol, butyric acid was added either gradually or at different growth phases of the fermentation. As a control, *C. pasteurianum* was cultivated on glycerol as the sole carbon source. In all fermentations, the pH was regulated at 6 by the addition of 5 N KOH.

**Fig. 2:**
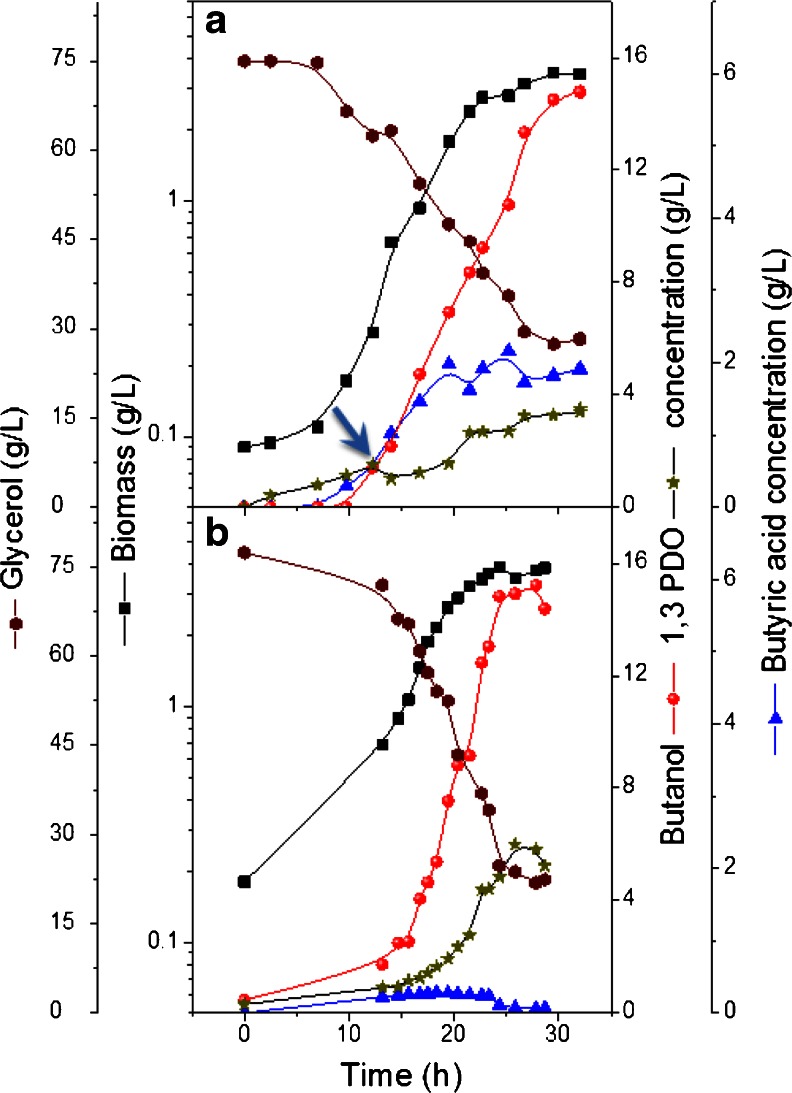
Growth, butanol and 1,3-PDO production in batch cultures of *C. pasteurianum* DSMZ 525 grown on glycerol fed with butyrate (**a**) or on glycerol (**b**). *Arrow* shows the beginning of the continuous butyrate addition

As can be seen in Fig. 2a, without butyrate addition, the major products formed during glycerol fermentation were *n*-butanol (15.2 g/L) and 1,3-PDO (6.2 g/L). The production of butyric and acetic acids was negligible during the whole fermentation and did not exceed 0.5 g/L. At slightly acidic pH of 6, butyric acid addition resulted in a slower growth rate. In fact, the addition of butyric acid at the beginning of the batch culture extended the lag period significantly and no growth was initiated if the initial pH was lower than 5.5 (data not shown). Butyric acid was then added either continuously (Fig. 2b) or at the mid and late logarithmic growth phases (growth OD of 6 and 9, respectively).

**Fig. 3:**
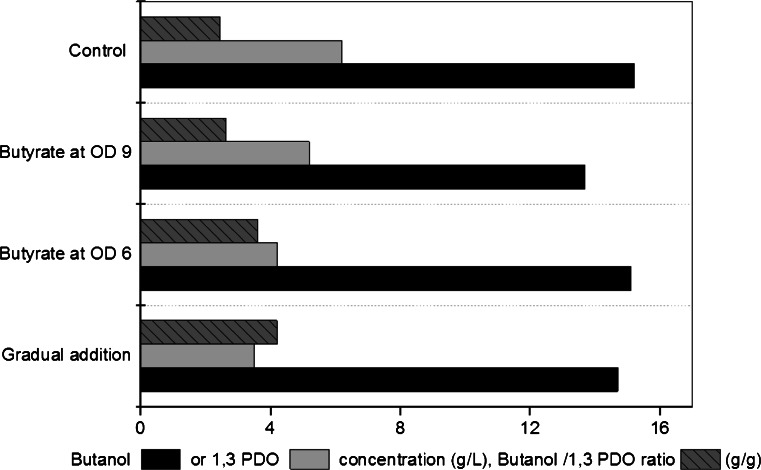
Comparison between concentrations of butanol, 1,3-PDO and their ratio as affected by the addition of butyric acid in pH-controlled batch cultures

Generally, the various strategies used to add butyric acid into the fermentation of glycerol did not result in an increase of *n*-butanol production. Moreover, the utilization of butyric acid as a co-substrate depends on the growth phase. Whereas acid addition at the late logarithmic phase (at biomass concentration of 3.1 g/L) stopped the bacterial metabolism and no butyrate consumption or *n*-butanol production occurred, the gradual addition during the fermentation (Fig. 2b) or at the mid logarithmic phase (at biomass concentration of 2.1 g/L, data not shown) resulted in significant acid consumption and a comparable *n*-butanol concentration as the control (Fig. 2a). More significantly, the addition of butyric acid had an influence on the 1,3-PDO formation and consequently on the butanol/1,3-PDO ratio (Fig. 3). Previously, we showed that a pH shift to the acidic range stopped 1,3-PDO production and increased the butanol/1,3-PDO ratio significantly (data not shown). A similar effect is demonstrated here with butyric acid addition, and the earlier the addition of butyric acid to the culture media, the less the 1,3-PDO is formed and the higher the butanol/1,3-PDO ratio that could be achieved (Fig. 3).

### The effect of glucose as co-substrate on bacterial growth and acid production

As shown in Fig. 1, mixed substrate fermentation with glucose in anaerobic bottles increased *n*-butanol production significantly. Moreover, since no natural pathway exist for 1,3-PDO synthesis from glucose, the butanol/1,3-PDO ratio increased drastically in such anaerobic bottles. Therefore, in pH-controlled bioreactors, we tested the effect of glucose addition as co-substrate in different ratios (glucose-to-glycerol ratio, 1:0, 1:1, 4:1 and 1:4). All the co-substrate fermentations were inoculated with cells grown on glycerol. Generally, increasing the glucose fraction in the co-substrate fermentation increased biomass and acid production significantly as can be seen from the base consumption in the pH-controlled experiments. The specific growth rate of the bacterium grown in the presence of glucose was generally higher and reached 0.25 h^−1^ (±0.02 at the different co-substrate fermentations) compared to 0.19 h^−1^ on pure glycerol.

**Fig. 4:**
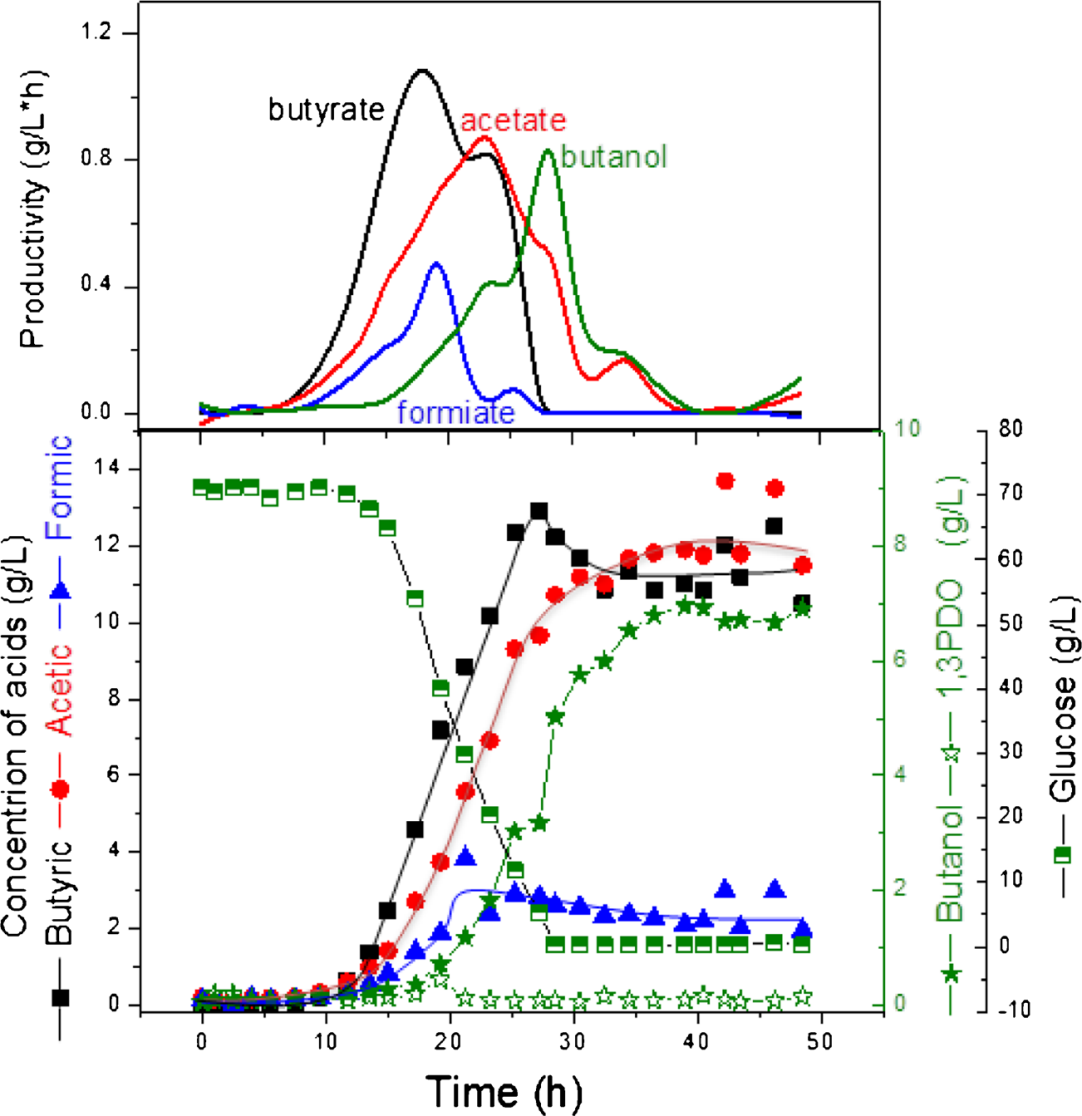
Product formation by *C. pasteurianum* during growth on glucose in a pH-controlled batch culture and their productivities

Growth on glucose as the sole carbon source is shown in Fig. 4. Being a classical acid producer, *C. pasteurianum* was known to ferment carbohydrates to butyrate, acetate, CO_2_ and H_2_ (Dabrock et al. 1992). A maximum *n*-butanol concentration of 6.9 g/L was obtained, and most of the glucose was directed for the production of organic acids. Moreover, unlike glycerol fermentation, the two phases, acidogenesis and solventogenesis, were clearly separated during glucose fermentation (Fig. 4).

### Different ratios of glucose and glycerol for the optimized *n*-butanol production in mixed substrate fermentation

**Fig. 5:**
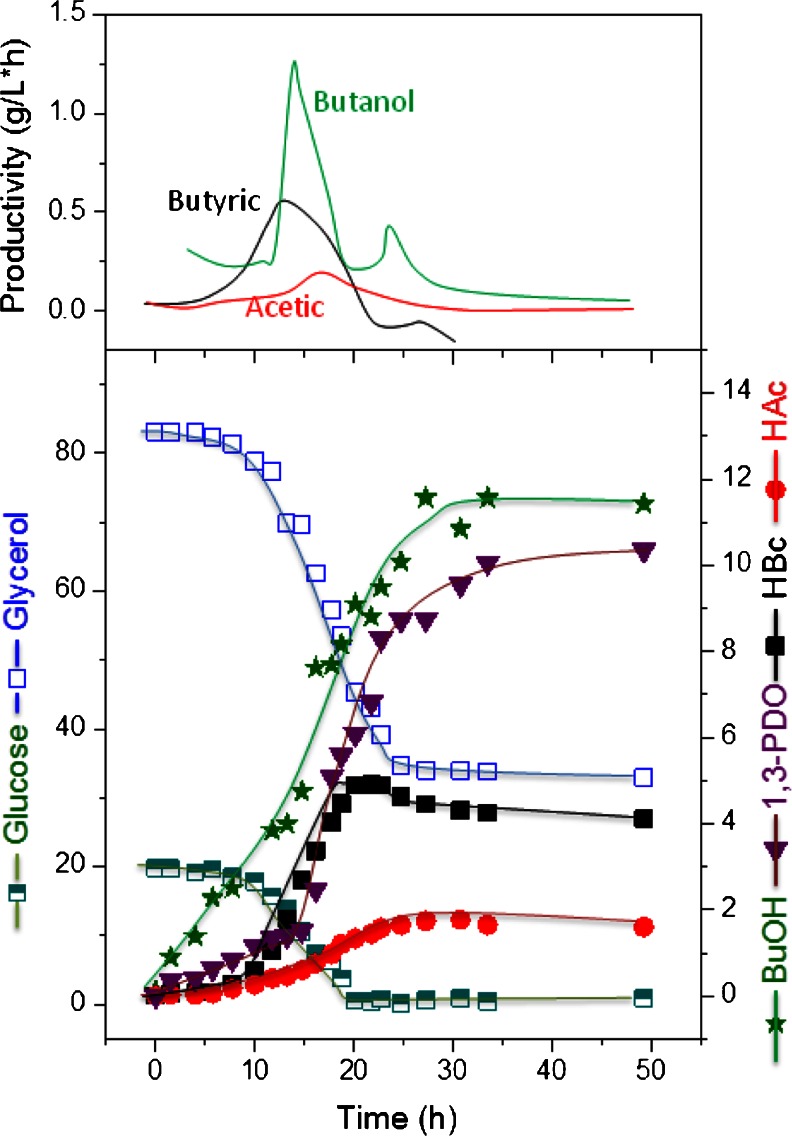
Product formation by *C. pasteurianum* DSMZ 525 during growth on glucose/glycerol blend in a ratio of 1:4 in a pH-controlled batch culture and productivities of the metabolites

**Fig. 6:**
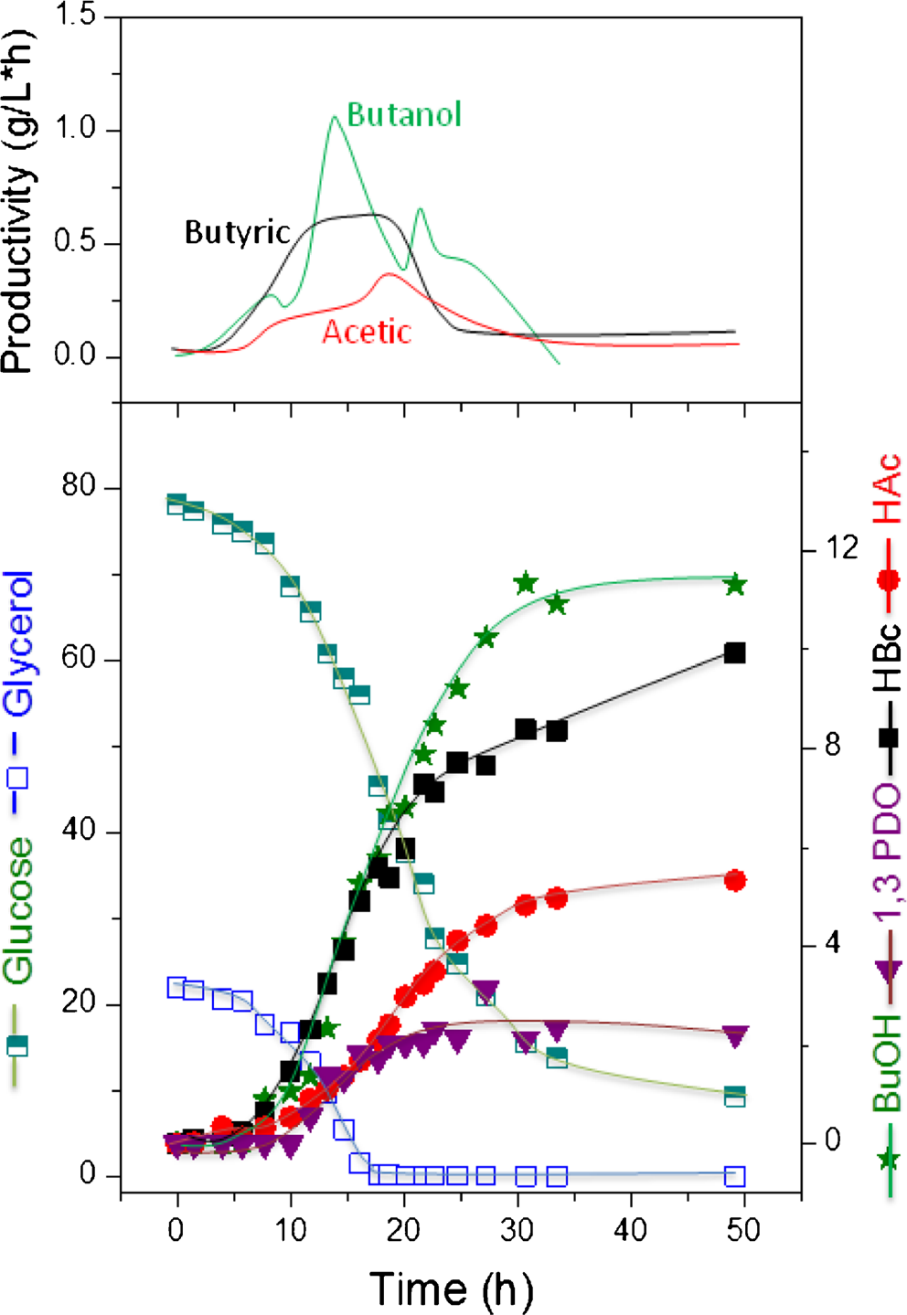
Major product formation by *C. pasteurianum* during growth on glucose/glycerol blend in a ratio of 4:1 in a pH-controlled batch culture and productivities of the metabolites

**Fig. 7:**
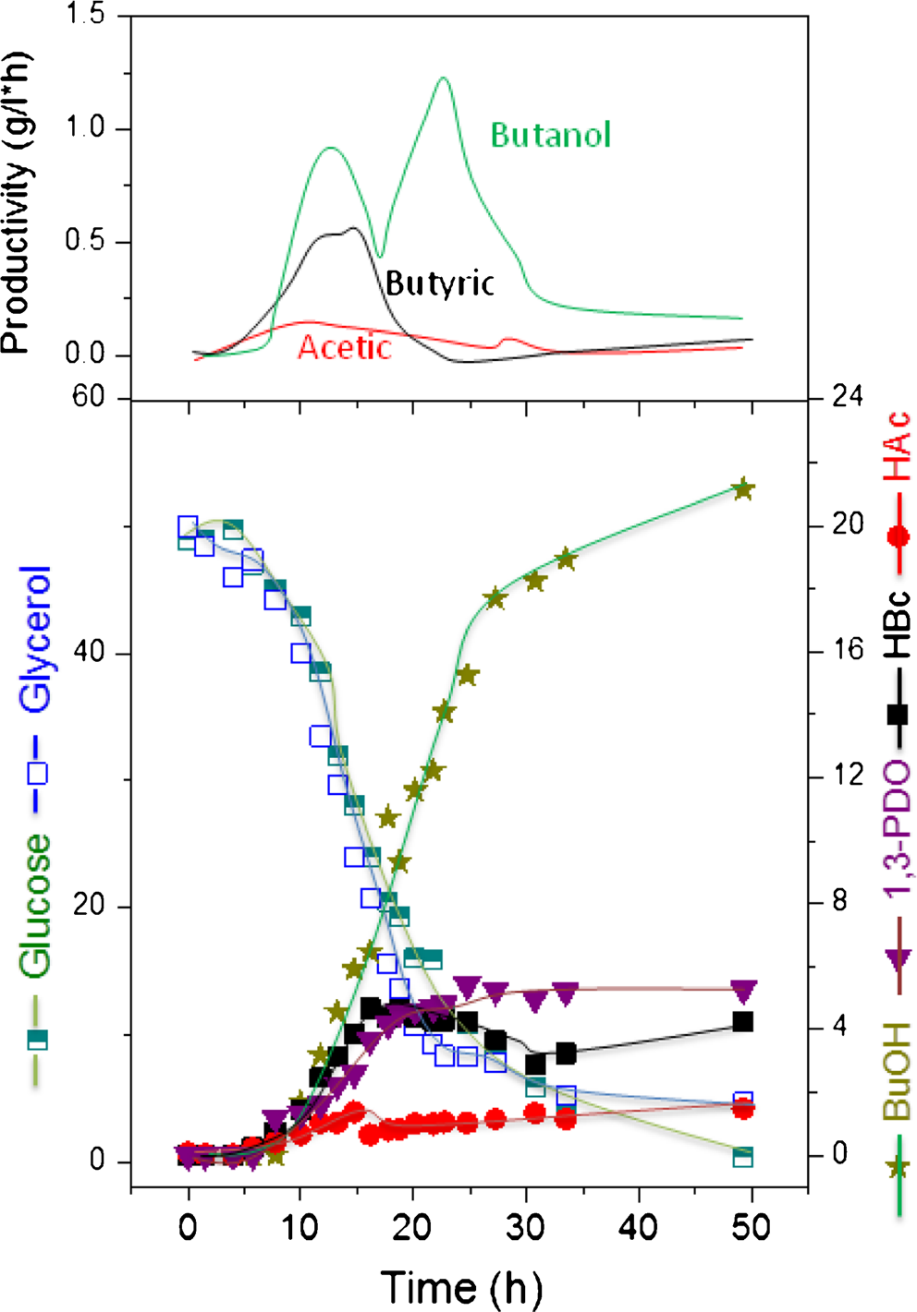
Time dependent fermentation profile of *C. pasteurianum* DSMZ 525 growing on glucose/glycerol blend in a ratio of 1:1 in a pH-controlled batch culture

Since glycerol is the natural substrate for 1,3-PDO formation in *C. pasteurianum*, the 1,3-PDO formation increased with increasing glycerol portion in the co-substrate fermentation (the 1,3-PDO production increased from 0 to 2.2, 5.2 and 10.3 g/L at glucose-to-glycerol ratio of 1:0, 4:1, 1:1 and 1:4). Moreover, the typical fermentation profile with two phases, namely, acidogenesis and solventogenesis observed in mono-substrate fermentation (Fig. 4b) was absent here and *n*-butanol was produced at an early stage in all the co-substrate fermentations (Figs. 5, 6 and 7).

As can be seen in Fig. 5 with a glucose-to-glycerol ratio of 1:4, immediately after glucose consumption, the glycerol consumption rate decreased from 4.3 to 2.3 g/L × h and lasted for 5 h and then stopped completely. A maximum *n*-butanol concentration of 11 g/L was obtained. On the other hand, in experiments with a glucose-to-glycerol ratio of 4:1 (Fig. 6), and after the onset of glycerol limitation, the *n*-butanol productivity decreased from 0.76 to 0.4 g/L × h, and a maximum *n*-butanol concentration of 12 g/L was obtained. At this glucose-to-glycerol ratio, most of the glucose utilized was directed to acids, the glucose consumption rate remained unaffected by the limitation of glycerol and the cells utilized glucose till the end of the experiment.

**Fig. 8:**
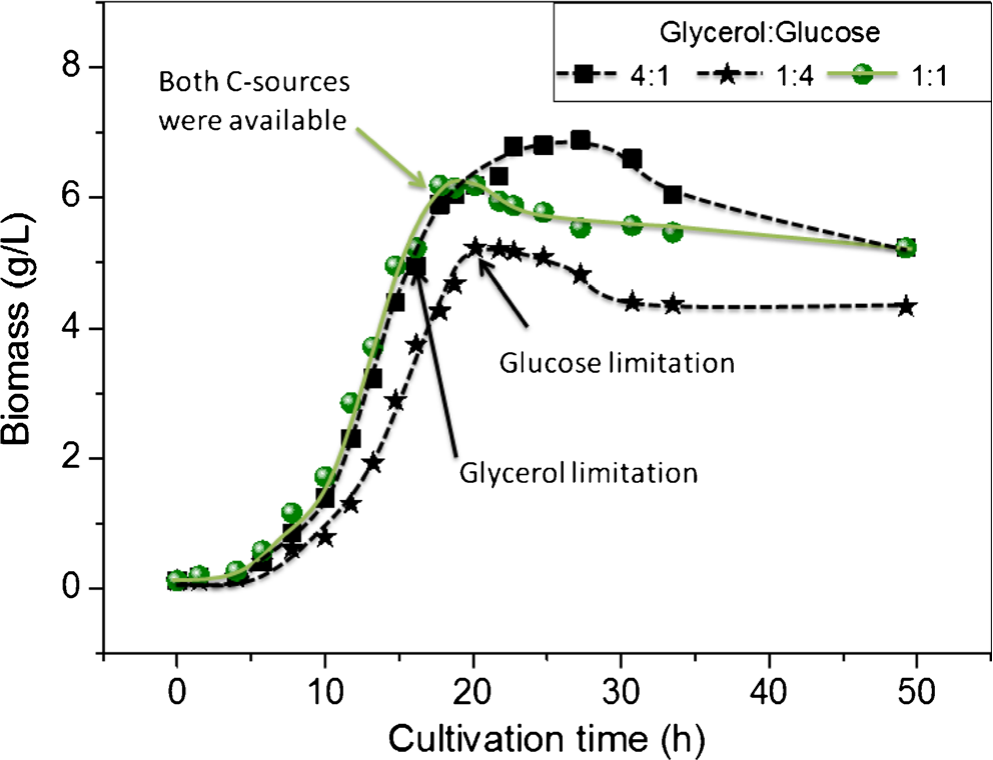
Biomass formation as affected by carbon source limitation in the mixed substrate fermentation

In the fermentation with glucose-to-glycerol ratio of 1:1, where no limitation of either substrate was observed, the maximum *n*-butanol concentration was obtained and recorded 21 g/L (Fig. 7). This is the highest concentration ever reached in literature of *n*-butanol with batch culture and without in situ removal of butanol. The maximum productivity of *n*-butanol recorded a value of 0.9 g/L × h. A comparison of the biomass formation as affected by different substrate limitation in the co-substrate fermentation is depicted in Fig. 8.

### Co-substrate fermentation using spruce BH and glycerol

Generally, substrates are a major proportion of product costs. Hence, to increase the economical feasibility of *n*-butanol production, we used BH instead of pure glucose in this experiment. The co-substrate fermentation was performed using diluted spruce BH. The hydrolysate was autoclaved separately and added to the sterile medium containing glycerol. Table 1 summarizes the results obtained with the hydrolysate in a ratio of 1:1 with glycerol and the results obtained at different ratios with pure glucose. A maximum *n*-butanol titer of 17 g/L was reached in the mixed substrate fermentation with BH. Remarkably, in the mixed substrate fermentation with BH and following the same trends showed in Fig. 8, the glycerol consumption stopped at the onset of glucose limitation.

**Table 1:** Mono- versus co-substrate fermentations using *C. pasteurianum* DSMZ 525

Mono/mixed substrate	Concentration (g/L)					Yield (*g*/*g*_C3+C6_)		Overall butanol productivity(g/L × h)
	Butyric acid	Acetic acid	1,3-PDO	Butanol	Biomass	Butanol	1,3-PDO	
Mono-substrate								
Glucose	10.5	11.9	0.0	6.9	13.2	0.1	0	0.21
BH	11.7	15.5	0.0	6.8	8.5	0.1	0	0.20
Glycerol	0.1	0.4	5.3	13.9	3.2	0.25	0.1	0.68
Mixed substrates								
Glu to Gly								
4:1	9.9	5.3	2.2	11.3	6.8	0.13	0.03	0.41
1:1	4.2	1.5	5.2	21.1	6.1	0.23	0.06	0.69
1:4	4.11	1.6	10.3	11.4	5.2	0.17	0.15	0.37
Mixed substrates								
BH to Gly								
1:1	4.1	4.2	1.8	17.4	6.1	0.2	0.02	0.62

*BH* biomass hydrolysate, *Glu* glucose, *Gly* glycerol

## Discussion

Despite the high potential of *C. pasteurianum* for the production of *n*-butanol (Biebl 2001; Malaviya et al. 2012; Khanna et al. 2013a, b), only a few publications have dealt with the optimization of *n*-butanol production with this microorganism. Previously, with *C. pasteurianum*, instability and great variation in the formation of the two major products 1,3-PDO and butanol was revealed after growth on glycerol (Biebl 2001). The author showed that under almost the same conditions, the strain could produce 1.3-PDO or *n*-butanol as the major product. Recently, many authors have optimized the cultivation conditions to favour the production of one of these two products (Moon et al. 2011; Khanna et al. 2013a, b). Obviously, both products are essential for the balance of the redox within the cell.

Previously, it was shown that acetate and butyrate addition triggered the pathway in *C. acetobutylicum* to shift from acidogenesis to solventogenesis (Li et al. 2011). Therefore, we investigated the effects of addition of different co-substrates on the production of *n*-butanol and 1,3-PDO by *C. pasteurianum* DSMZ 525. At a constant initial pH of 7, in anaerobic bottles, the results obtained, with respect to acid addition, were in agreement with the work done by Li et al. (2011) for *C. acetobutylicum* and by Kao et al. (2012) for *C. pasteurianum* CH4 strain. The specificity of *n*-butanol formation by *C. pasteurianum* DSMZ 525 was also enhanced significantly by the addition of acids (Fig. 1). It should be stressed here, that in *C. pasteurianum* grown on glycerol, where both acid and solvent formation occurs simultaneously, the positive effect of acid addition remains unclear (Figs. 1 and 2). On the other hand, in pH-controlled reactors at pH = 6, the addition of butyrate did not enhance *n*-butanol production significantly (Fig. 2). Since pH is known to be a crucial factor affecting the performance of ABE fermentation by Clostridia (Dabrock et al. 1992; Haus et al. 2011; Moon et al. 2011; Malaviya et al. 2012; Khanna et al. 2013a; Millat et al. 2013), the variation of pH in anaerobic bottles may explain such difference. In the anaerobic bottle experiments shown in Fig. 1, the buffering agent (CaCO_3_) is not sufficient to stabilize the pH, and ‘acid crash’, a phenomenon occasionally occurs in pH-uncontrolled batch fermentations, will indeed affect the growth and product formation (Maddox et al. 2000). Indeed, the varied pH and the accumulation of undissociated acids in broths may then explain the premature cessation of metabolism and glycerol consumption in batch cultures. Additionally, the pressure created in such anaerobic bottles resulted in the dissolution of the produced gas into the medium, and therefore adding unstudied factors inside the anaerobic bottles. For example, it was shown that culture grown for 4 days in standard bottles with pressurized cultivation consumed 15 g/L glycerol compared to 44 g/L in bottles with regular pressure release. Moreover, *n*-butanol production increased significantly in the unpressurized bottles and reached 6.7 g/L compared to 1.6 g/L in standard bottle with pressurized cultivation.

In the pH-controlled experiments, and since butyric acid was added at the same concentration, the effect of the undissociated acids were shown to be dependent on the growth phase. This explains the diminished growth rate or even the inhibition of growth after the addition of butyric acid at a fixed pH value, or the extended lag period or growth failure if butyric acid was added at the beginning at pH set value of 6 or 5.5, respectively. Interestingly and as shown in Figs. 1 and 3, butyric acid addition resulted in a higher butanol/1,3-PDO ratio. Acetone was never detected in samples taken at the experiment end. This fact excludes the possible redirection of butyrate to *n*-butanol pathway by the CoA-transferase linked to acetone production. Previously, it was reported that iron excess conditions enhanced the butanol/1,3-PDO ratio significantly in *C. pasteurianum* (Dabrock et al. 1992; Jensen et al. 2012a). Moreover, in *C. pasteurianum* batch culture controlled at pH 6, a pH shift to the acidic range at the mid logarithmic phase stopped 1,3-PDO formation but enhanced *n*-butanol productivity significantly (data not shown). Still the exact mechanism of the positive effect of butyric acid addition on *n*-butanol production in *C. pasteurianum*, especially in pH-uncontrolled cultures, is not known and need more investigation.

Triggering bacterial own acid production through glucose addition, however, enhanced biomass and *n*-butanol production significantly. Using glucose as a co-substrate, the butanol/1,3-PDO ratio increased significantly. In fact, the 1,3-PDO concentration increased in *C. pasteurianum* cultures with the increase in the glycerol moieties of the co-substrate fermentation (Table 1). On the other hand, acid production increased significantly with the increase in the glucose moieties of the co-substrate fermentation (Table 1). Acetate and butyrate are produced from acetyl-CoA and butyryl-CoA, respectively, by means of two analogous steps, which result in the generation of one ATP molecule per each reaction, and this explain the increase of biomass observed with the increase in acid production in the co-substrate fermentation (Table 1).

For *n*-butanol production by *C. pasteurianum* DSMZ 525, the limitation of either substrate during the course of fermentation should be avoided (Fig. 7). Experiments with glucose or glycerol limitation showed a decrease in the *n*-butanol production significantly (Figs. 5 and 6). The effect of mixed substrate fermentation on *n*-butanol production appears to be strain dependent. In *C. acetobutylicum*, *n*-butanol productivity was much lower in mixed substrate fermentation than on glucose, and a maximum productivity of 0.42 g/L × h compared to 0.9 g/L × h was reported on mixed or mono-substrate fermentation, respectively (Andrade and Vaconcelos 2003). On the other hand, *C. pasteurianum* strain *CH4* produced 13.2 g/L *n*-butanol with a productivity of 0.19 g/L × h in co-substrate fermentation compared to 11 g/L and 0.14 g/L × h on glycerol (Kao et al. 2012).

To compete effectively with petrochemical-derived butanol, the use of lignocellulosic biomass as a substrate has a great potential. Compared to pure glucose, the growth rate on BH is slightly slower (0.2 compared to 0.25 h^−1^ with pure glucose). The maximum *n*-butanol productivities were almost similar in glucose or BH in mono-substrate or mixed substrate fermentation with glycerol (Table 1). Interestingly, in experiments with 1:1 ratio of BH to glycerol, glucose was consumed with a rate of 3.2 g/L × h compared to a glycerol consumption rate of 2.2 g/L × h. In agreement with the results shown in Fig. 5, with low glucose-to-glycerol ratio, inhibition of glycerol utilization was noticed shortly after glucose limitation.

Although *C. pasteurianum* can utilize either glycerol or glucose separately as a sole carbon and energy source, the observed inhibition of glycerol utilization after the exhaustion of glucose is still not understood in the mixed substrate fermentation by *C. pasteurianum*, and is therefore crucial if an optimization of *n*-butanol production is aimed. The growth of the bacterium was also stopped after glucose limitation (Fig. 8). This was not found when glycerol was limited in fermentation with a high glucose/glycerol ratio (Fig. 8). One explanation for this may be the improved acclimation of *C. pasteurianum* cells to *n*-butanol stress in the presence of glucose, which resulted in a higher resistance to *n*-butanol toxicity. Indeed, the tolerance of the bacteria to higher *n*-butanol concentrations was significantly enhanced when both substrates were simultaneously fermented in anaerobic bottles compared to mono-substrate fermentation with glycerol (data not shown). The production of acids during glucose utilization plays an important role in maintaining the redox balance of the cells by oxidizing NADH to NAD+. With mixed substrate fermentation, in *C. butyricum* (Saint-Amans et al. 2001) and in *C. acetobutylicum* (Vasconcelos et al. 1994), it was shown that most of the reduced ferrodoxin produced by the pyruvate ferrodoxin oxidoreductase was used to generate NADH leading to low hydrogen production. Moreover, the improved *n*-butanol tolerance of the cells might be associated with the energetic status of the cell in the presence of glucose. Acid production parallels the energy production necessary for cell viability and homeostasis at high *n*-butanol concentration. This may explain the enhancement of culture stabilities in *C. acetobutylicum* and prevention of cell degeneration in the mixed substrate fermentation (Andrade and Vaconcelos 2003). Although a number of possible mechanisms may account for this, further work is required to determine the underlying mechanism(s).
